# The Relationship between Religion, Substance Misuse, and Mental Health among Black Youth

**DOI:** 10.3390/rel14030325

**Published:** 2023-02-28

**Authors:** Camille R. Quinn, Bernadine Waller, Ashura Hughley, Donte Boyd, Ryon Cobb, Kimberly Hardy, Angelise Radney, Dexter R. Voisin

**Affiliations:** 1 Center for Equitable Family & Community Well-Being, School of Social Work, University of Michigan, Ann Arbor, MI 48109, USA; 2 Department of Psychiatry, Columbia University Irving Medical Center, New York, NY 10032, USA; 3 College of Social Work, The Ohio State University, Columbus, OH 43210, USA; 4 School of Social Work, Rutgers, The State University of New Jersey, New Brunswick, NY 08901, USA; 5 School of Social Work, Fayetteville State University, Fayetteville, NC 28301, USA; 6 School of Applied Social Sciences, Case Western Reserve University, Cleveland, OH 44106, USA

**Keywords:** black youth, religiosity, mental health, critical theory

## Abstract

Studies suggest that religion is a protective factor for substance misuse and mental health concerns among Black/African American youth despite reported declines in their religious involvement. However, few studies have investigated the associations among religion, substance misuse, and mental health among Black youth. Informed by Critical Race Theory, we evaluated the correlations between gender, depression, substance misuse, and unprotected sex on mental health. Using multiple linear regression, we assessed self-reported measures of drug use and sex, condom use, belief in God, and religiosity on mental health among a sample of Black youth (N = 638) living in a large midwestern city. Results indicated drug use, and sex while on drugs and alcohol, were significant and positively associated with mental health symptoms. Belief in God was negatively associated with having sex while on drugs and alcohol. The study’s findings suggest that despite the many structural inequalities that Black youth face, religion continues to be protective for Black youth against a myriad of prevalent problem behaviors.

## 1. Introduction

Despite a decline in formal religious involvement in the United States, Black American youth are the most religious relative to their other racial and ethnic peers (Diamant 2018; Mohamed et al. 2021; Pew Research Center 2018). Black (or African American, we use the terms interchangeably; Slopen et al. 2010) youth tend to be affiliated with historically Black Christian congregations, and self-report high levels of biblical literalism. One study noted that 95% of African American youth “report a belief in God,” while 86% “believe in a judgement day” (Christerson et al. 2010, p. 119). According to the Pew Research Center, which tracks religious involvement among youth and adults, 60% of people associated with historically Black Protestant churches believe the Bible is the actual word of God (Miech et al. 2019; Pew Research Center 2009). Nearly two-thirds (64%) of Black youth are highly religious, routinely attending church services and privately praying, while just over one-third (39%) of other racial groups report the same (Pew Research Center 2018). Further, 61% of Black youth reported praying at least once daily, while only 39% of their peers noted similarly (Diamant 2018).

Religious involvement is often termed religiosity, the practice of one’s religion and is conceptualized as the involvement in institutions that allow for formal expression with the sacred (Dill 2017). It includes a fundamental belief in God and is measured by prayer, church attendance, and meditation frequency. For example, some scholars have found that research focused on the role of stress within a stress process model has shown that individual religiosity buffers the adverse association between financial strain and mental health (Upenieks and Ellison 2023). Recent trends suggest declines in Black youth religiosity, especially during the last two years of high school when many become disinterested in formal religious involvement and stop attending religious services regularly. Specifically, higher levels of attendance at religious events, as well as the endorsement of the importance of religion for Black versus white 12th graders has decreased. Of note, the proportion of Black compared with white 12th graders who attended religious services at least once a week and consider religion to be important in their lives was about 20% smaller in 2012 versus 2006 (Child Trends 2014; Miech et al. 2019).

The decrease in religiosity among Black youth could be related to what some scholars refer to as a potential convergence in the Black versus non-Black gap in substance misuse, i.e., marijuana use (Miech et al. 2019), and other behaviors such as risky sex or sex positivity. Few studies have explored contextual factors such as substance misuse and sex and religiosity, including different aspects of religious involvement across a single sample of Black youth. Consequently, what we know about these associations are scattered across studies and have focused on adults (Chen and VanderWeele 2018; Upenieks and Ellison 2023). Thus, the aim of this study is to investigate the relationship between religiosity and drug use and sex, and mental health symptoms among Black adolescents who reside in low-income communities.

## 2. Background/Literature Review

### 2.1. Black Youth, Spirituality and the Church

It has been posited that a stronger sense of personal religiosity could help individuals focus on having a deep spiritual life and placing less emphasis on their external circumstances, which might serve as a protective factor in life (Upenieks and Ellison 2023). Black youth largely maintain religious involvement at similar rates to their parents’ (Diamant 2018). Thus, Black youths’ overall high religiosity might reflect a reliance upon their faith in God or a higher power during difficult times, similar to their parents’ practices. Spirituality has been used to examine how youth have positive outcomes despite negative experiences with their families, friends, their schools, communities, or society (Edwards and Wilkerson 2018; Hardy et al. 2019; Salinas et al. 2018). Notably, Black youth due to structural inequalities and poverty (Quinn et al. 2019) experience greater adversity compared with their peers from other races and ethnic groups, even though they make up only 14% of the United States youth population (KIDS COUNT Data Center n.d.a). Specifically, 32% of Black youth living in the United States live below the poverty level (KIDS COUNT Data Center n.d.b), and of them, 40% have had two or more adverse childhood experiences (Child and Adolescent Health Measurement Initiative 2016). Such adverse experiences may undermine their physical, mental, and spiritual well-being.

As previously noted, religion might be protective for Black youth (Holland 2016; Voisin et al. 2016b). Crawford Sullivan and Aramini (2019) contend that religion promotes resilience to buffer risky behaviors, especially for youth of color. Hardie et al. (2016) suggests attending religious services is particularly helpful for Black youth because the result is social capital. Further, the relationship between religious organizations and community involvement prepares youth for obstacles they could face and provides a refuge from daily stressors and adversity (Edwards and Wilkerson 2018; Kim and Esquivel 2011). Edwards and Wilkerson (2018) note that church and/or religious-based groups help support Black youths’ mental well-being. Specifically, Black churches sustain low-income families through religious rituals that maintain social networks and provide emotional support (Steers et al. 2019). Churches serve as a liaison for community-based programs that support the development of Black youth (Ceasar et al. 2017). Further, religious organizations that host networking activities for youth, and organize school and other advocacy efforts such as community protests (Rhoads 2016) positively benefit them. Given the mixed evidence about the role of the church and spirituality for Black youth in promoting coping strategies for social obstacles they face (Pascaru 2019); more research is needed to better understand its role in the social and emotional well-being of Black youth (Rose et al. 2020; Rose et al. 2021).

### 2.2. Black Religious Involvement and Resiliency

Spirituality has been used to unify and strengthen individuals who live in low-income communities (Dill 2017). Furthermore, overwhelmingly Black houses of worship have long been a stalwart and hub of the Black community. Ongoing structural and systemic barriers have largely precluded Black Americans from readily accessing mainstream resources afforded to whites (Bailey et al. 2017). Parishioners of Black houses of worship responded by expanding their reach to include healthcare promotion and delivery, social and political organizing, educational and childcare services, and information vital for the community’s overall well-being (Hankerson et al. 2015; Harris-Lacewell 2007). The church has been a respite for youth experiencing various challenges related to mental and sexual health, and substance misuse (Breland-Noble et al. 2015; Boyd et al. 2022b; Voisin et al. 2016b). Despite advances in understanding Black youth’s reliance upon their faith in God and religious resources, scholarship about the role of religion, and mental and sexual health behaviors across a single population of Black youth is scant. This study seeks to close the gap with its focus on Black youth in largely low-income communities.

### 2.3. Black Youth and Mental Health

Although efforts to address mental health concerns in the United States exist, reducing and/or eliminating racial disparities in mental health outcomes has been slow (Alvidrez and Barksdale 2022). Research utilizing numerous theoretical frameworks and mixed results on the relationship between spirituality/religion (S/R) (e.g., spiritual coping) and youth mental health indicate positive effects on African American health attitudes, as well as youth behaviors (Cotton et al. 2006; Price-Spratlen 2015), and improved life satisfaction (Taylor et al. 2014). In addition, scholars noted increased psychological health, and prosocial behavior, as well as a negative relationship to substance misuse and early sexual debut (Breland-Noble et al. 2015; Chiswick and Mirtcheva 2013). These study results provide significant consideration for integrating spirituality into social work practice with African American youth (Belcher and Mellinger 2016).

Black youth receive significantly less evidence-based mental health assessments and treatments than other racial groups and are more likely to receive services in a non-specialty care facility such as foster care or youth detention (Center for Behavioral Health Statistics and Quality 2021; Hoge et al. 2022). In addition, structural racism, and a lack of cultural sensitivity by service providers often creates barriers to quality mental health care. Such misalignments often expose African American youth to the full brunt of environmental stressors (Brenner et al. 2013; Lee and Crooks 2021; Snowden 2012) that can exacerbate mental health disparities, including depression (Auguste 2022; Auguste et al. 2021; Brown et al. 2006; Freeny et al. 2021), and race-based traumatic stress (Carter 2007; Jernigan and Daniel 2011). Two interrelated barriers contributing to the limited use of specialty care are the prevalent use of alternative coping skills, i.e., S/R (Caldwell et al. 2016), and perceived discrimination (Smith and Nicholson 2022). Implementing culturally adapted mental health interventions, including complementary faith-based approaches, may create a climate promoting an awareness of limited mental healthcare access among Black youth. An analysis of structural factors contributing to mental health disparities may activate change agents to participate in structural change to systems that maintain racism and discrimination, negatively impacting Black youth mental health and well-being.

### 2.4. Black Youth and Substance Misuse

Understanding the relationship between religion and substance misuse among Black youth is key to assessing their association(s) with mental health and sexual health behavior(s). Christerson et al. (2010) noted a sample of African American youth reported the highest levels of pressure to engage in sexual activity from their love interests and friends. Despite this fact, most African American youth in the study sample did not report having sex, with only 28% reporting they engaged in sexual activity (Christerson et al. 2010). In a study with Black girls in a southern detention facility, scholars noted that younger girls were more likely to engage in sex if they used drugs and alcohol (Quinn et al. forthcoming a). However, this study did not include S/R as a factor. However, several reviews, including a meta-analysis and a systematic review established that S/R reduces the risk of substance misuse (Chitwood et al. 2008). Regnerus et al. (2003) notes prudent accounts of youths’ S/R requires attention to intergenerational social bonds, changing family structures and community norms, which are needed to develop more sophisticated instruments that measure S/R (Chitwood et al. 2008), and to provide evidence of future interventions’ efficacy in helping people with substance misuse problems (Hai et al. 2019). However, more research needs to be conducted to broaden the literature base in this area, especially for Black youth to build upon the work that has already been done (Chitwood et al. 2008).

Chitwood et al. (2008) reported on 105 studies about S/R. The samples comprised youth and adults, as well as Black/African Americans though the studies with Black youth were not disaggregated by race (Nasim et al. 2006; Steinman and Zimmerman 2004). Studies about youth comprised 10 measures of spirituality such as religious affiliation (Marsiglia et al. 2005), religious service(s) attendance (McBride et al. 1996), and family religiosity (Merrill et al. 2001). Hai et al.’s (2019) review of 20 intervention studies suggested an age range between 17.6 and 49; seven of those studies mainly included Black or Latino/a/Hispanic participants. For example, some interventions with African American youth included spirituality-modified cognitive behavioral therapy and 12-step-oriented interventions (Harvey and Hill 2004; Stoltzfus 2007). Kelly et al. (2015) also conducted a meta-analysis with 62 studies over four decades, including 145 effect sizes from 193,656 adolescents about their S/R and delinquency (alcohol use, illicit drug use, and nondrug delinquency). Overall, they confirmed that religious involvement is negatively related to delinquent behaviors, i.e., alcohol and illicit drug use, regardless of the measurement characteristics. However, two studies reflected what some might call a weakening of religiosity. One study conducted in the 1960s had a larger effect size (i.e., more negative) than a survey conducted in the 2000s. Thus, as the percentage of African American students increased throughout studies, less relationship was found between alcohol use and church attendance. This finding is inconsistent with the previous result that religious influence is more likely among racial and ethnic groups, especially Blacks (Diamant 2018; Kelly et al. 2015; Mohamed et al. 2021; Pew Research Center 2018). The gaps identified in these reviews suggest that more empirical investigations of S/R and other important factors among Black youth are warranted to determine the factors that are associated with improved mental health outcomes.

### 2.5. The Current Study

Some research suggests that religion could have even stronger health effects on children at younger ages (Dupre et al. 2006; Idler 2014). Current evidence indicates that adolescents’ religious involvement could protect against certain adverse behaviors and promote positive behaviors (Nonnemaker et al. 2003; Rew and Wong 2006). The current study investigates the relationship between religiosity, drug use and sex, and mental health symptoms among Black adolescents who live in low-income communities. Therefore, within the current study context, we seek to examine how a sample of Black youth endorse accounts of their mental health experiences given the multiple intersections of their gender, SES, and their sexual behavior.

Black youth are more likely to experience both health and mental health issues even though research on their rates of substance misuse suggests they are lower than those reported by their peers in other racial and ethnic groups, especially white youth (Child Trends 2014; Miech et al. 2019). Furthermore, there has been limited consideration and theorization of Black life, including Black youth’s spirituality that has been linked with racism (Alhassan 2020). Consequently, this study is informed by Critical Race Theory (CRT) to frame the effects of race always present in American society and to illuminate the importance of focusing on a Black sample of youth (Abrams and Moio 2009; Quinn and Grumbach 2015). CRT tenets include: (1) endemic racism; (2) race as a social construction; (3) differential racism; (4) interest convergence/materialistic determinism; (5) voices of color; and (6) antiessentialism/intersectionality. Investigating different aspects of an individual’s behavior should be considered with other important factors to understand their mental (and sexual) health behaviors and outcomes. Using CRT as an overarching lens allows us to understand society’s oppressive aspects to engage in micro- and macro-level transformation and contextualize our study findings (Solorzano and Bernal 2001). We propose two primary hypotheses: (1) that having sex while on drugs and alcohol correlates negatively with mental health, and (2) that religiosity would be positively associated with better mental health.

Below, we describe the data, methods, analytical approach, and results. We present the quantitative data analysis and conclude by synthesizing the results within the CRT framework to offer more nuanced insights into the empirical relationships between religion, sex, and substance misuse and Black youth’s mental health.

## 3. Materials and Methods

### 3.1. Data

The data for this study come from the parent study, the Resilience Project collected in 2013–2014, a study examining the risk and protective mechanisms related to sexual behaviors of Black adolescents living in four urban neighborhoods of concentrated poverty in Chicago: Englewood, Woodlawn, Kenwood, and South Shore. Youth were recruited from three high schools, one youth church group, two community youth programs, and four public venues (e.g., parks and fast-food restaurants). The response rate for this study was 87%, and the total participants were 548 Black adolescents, ages 12 to 17. These participants were recruited from low-income communities consisting predominantly of Black residents where the average annual median income ranged from $24,049 to $35,946, which is below the Chicago city average of $43,628. The percentage of single mother households in these areas ranged from 28.9 to 32.3%, with the city average being 13.9%.

To recruit adolescents, flyers about the study were posted at schools, community programs, and churches. The school principals, and leaders of church groups and youth programs, permitted the researchers to recruit participants for the study. Participants were required to have both parental consent (permission) and youth assent to enroll in the study. Youth recruited in public venues were only asked to participate if a parent was present to provide consent. Youth who returned consent forms signed by a parent or guardian were also enrolled in the study. Trained research assistants introduced the study to all potential participants recruited from the locations with a detailed letter describing the study along with parental consent and youth assent forms.

Participants recruited from schools, community programs, and churches were given a questionnaire at those respective locations. Youth who were recruited in public venues were given questionnaires in quiet spaces at or near those venues. In such instances, questionnaires were only administered to youth if a parent or a guardian was present to provide consent, and the questionnaire could be immediately issued. The questionnaire took approximately 45 min, after which the youth participant was given $10 cash compensation. The University Institutional Review Board of the last author who collected the data approved the study.

### 3.2. Measures

#### 3.2.1. Dependent Variables

*Mental Health* is the outcome variable for this study. It was assessed using the Brief Symptom Inventory (Derogatis and Kathryn 2000; Voisin et al. 2016a), which contains 18 items about mental health symptoms during the past seven days (e.g., nervousness or shakiness inside, spells of terror or panic, thoughts of ending your life). Response options were based on a five-point scale (not at all, a little bit, moderately, quite a bit, or extremely). A composite mental health score was calculated by summing the responses for the 18 items. Cronbach’s alpha was α = 0.92 (range 0 to 61).

#### 3.2.2. Independent Variables

Several variables made up religious activities. *Religiosity* was measured using a three-item, 4-point Likert type scale, with values ranging from 1 (never) to 4 (very often), with higher scores indicating higher participation in religious activity. A sample item included “In an average month, how often do you pray or meditate.” The Cronbach alpha for this measure is 0.72. *Religious Service Attendance* was measured using a single item, a 4-point Likert type scale, with values ranging from 1 (mother) to 4 (mother and father). Participants were asked the following question “Gone to a religious service or church-related event.”

Several variables measured substances “drug use and sex under the influence of drugs or alcohol.” *Drug Use* was assessed using three or more recreational drugs: “Have you taken a substance such as cigarettes, ecstasy, codeine, alcohol, and marijuana in the last 30 days?” *Sex while on Drugs or Alcohol* was assessed using the following question: “The last time you had sexual intercourse, did you have any alcoholic drinks and/or take any drugs before having sexual intercourse?” These dichotomous (i.e., *yes*, or *no*) variables were derived from previous studies (Boyd et al. 2021; Quinn et al. forthcoming a).

Several contextual variables were collected and included as covariates. Participants were asked to indicate their *Gender*, *Grade Level*, and *Condom Use*. *Gender* was assessed using a single item based on a dichotomous response of 0 (male) and 1 (female). *Grade Level* was evaluated using the following measure with the following question: “What grade level are you currently in,” which was based on a categorical variable (i.e., first-year, second-year student) response. *Condom Use* was included as a covariate based on a previous study finding from a moderation analysis and found that the interaction plot showed an enhancing effect such that as condom use increased, and depression among Black girls increased (Waller et al. 2022). We assessed *Condom Use* using the following question: “The last time you had sexual intercourse, did you use a condom?” that was based on a dichotomous (i.e., *yes*, or *no*) response.

#### 3.2.3. Analytical Strategy

All analyses were conducted on observations that included less than 5% missing data for the outcome variable, Mental Health. Table S1. presents demographic statistics among the study variables. Table S2. presents drug use variables. Bivariate correlation analysis was conducted on all study variables—outcome variable: Mental Health; and predictor variables: Sex while on Drugs and Alcohol, Drug Use, Religiosity, Religious Activities, and covariates: Gender, Grade Level, and Condom Use (Table S3). Lastly, we conducted a multiple regression analysis, including all the study predictors and covariates on the study outcome: Mental Health (Table S4). Survey data were analyzed based on listwise deletion. For survey scales, a mean score of the scale items was generated for participants with non-missing data. All analyses were conducted in STATA 17.

## 4. Results

### 4.1. Descriptive Results

Table S1 provides descriptive statistics for study variables (N = 637). The average age of the sample was 16 years (*SD* = 1.41) and individuals who self-identified as a girl or female made up 54% of the sample. First, approximately 20% of the sample reported Mental Health symptoms. Ninety-three percent of youth reported they Believe in God and 26% of youth reported that their S/R beliefs are very important. Lastly, 29% of young people stated that they prayed “every once in a while” and 40% reported attending church or other religious services “every once in a while.” Eighty-eight percent of the sample reported not having Sex while on Drugs and Alcohol and 40% reported marijuana use (Table S2).

### 4.2. Bivariate Correlations

Correlation results revealed that Sex while on Drugs and Alcohol was positively associated with Mental Health (*r* = 0.11, *p* < 0.05) (see Table S3). Drug Use was positively related to Mental Health (*r* = 0.18, *p* < 0.001) and Sex while on Drugs and Alcohol (*r* = 0.30, *p* < 0.001). Religiosity was negatively associated with Mental Health and positively associated with Drug Use (*r* = 0.11, *p* < 0.010). Church Attendance with parents was negatively associated with Religiosity (*r* = −0.25, *p* < 0.001). Gender was positively related to Mental Health (*r* = 0.13, *p* < 0.05), but was negatively associated with Sex while on Drugs and Alcohol (*r* = −0.15, *p* < 0.001), Drug Use (*r* = −0.17, *p* < 0.001), and Religiosity (*r* = −0.14, *p* < 0.001). Grade Level was positively associated with Mental Health (*r* = 0.14, *p* < 0.010) and negatively associated with Church Attendance (*r* = −0.12 *p* < 0.05) and Gender (*r* = −0.08, *p* < 0.001). Lastly, Condom Use was negatively associated with Mental Health (*r* = −0.13, *p* < 0.05).

### 4.3. Multiple Regression Analysis

Multiple linear regression was conducted between the outcome variable, Mental Health, predictor variables, and covariates. The overall model was statistically significant (F (11,298) = 5.661, *p* < 0.001), with an R^2^ of 0.17. Our results indicated that being female was associated with positive Mental Health (β = 0.16, *p* = 004), and Drug Use was statistically significant and positively associated with Mental Health (β = 0.24, *p* < 0.001). Surprisingly, Religiosity was negatively related to Mental Health (β = −0.12, *p* = 0.024). Church Attendance (i.e., service, or related event) was also negatively associated with Mental Health for Black youth (β = −0.12, *p* < 0.031). Black youth in the 10th (β = −0.14, *p* < 0.001) and 12^th^ (β = −0.18, *p* < 0.001) grades were associated with negative Mental Health. Lastly, Condom Use was associated with Mental Health (β = −0.13, *p* < 0.010).

## 5. Discussion

This paper examines different aspects of Black adolescent’s religious involvement and their behavior to assess their mental health and well-being. We noted significant relationships between mental health and Black youth who had sex while on drugs and alcohol, including 20% who reported mental health symptoms (i.e., suicidal thoughts, nervousness, panic spells), and 12% who reported having sex while they were on drugs and alcohol, respectively. This finding raises interest in the role of substances (i.e., drugs), given that studies about drug use among Black youth produce mixed results (McGee and Foriest 2009; McGee et al. 2018; Quinn et al. forthcoming a; Welch-Brewer et al. 2011). In the context of CRT, Black youth could be influenced by the overall quality of life they experience in low-income neighborhoods. The third CRT tenet, *differential socialization* suggests that individuals in power can racialize groups of people in different ways at different times based on social, historic, or economic need (Abrams and Moio 2009; Quinn and Grumbach 2015). Specifically, racism and power structures are used to reinforce concentrated disadvantage within minority communities, especially where Black youth reside. Racism is quite prevalent in American society, across social domains—individual, family, peers, school, community/neighborhood, as well as structural and systemic patterns of discrimination (Boyd et al. forthcoming). Our investigation of the relationships between religiosity and drug use and sex while on drugs and alcohol, and mental health symptoms among Black adolescents in a CRT context also reflects the first tenet—racism is endemic. Specifically, *endemic racism* suggests that Black youth in these low-income communities may be more prone to poor mental health given the structural aspects of poverty and racism, and their impact. Recent increases in suicide among Black youth could be evidence of this, along with the convergence of decreases in religiosity and potential increases in substance misuse (Boyd et al. 2022b; Lindsey et al. 2019; Quinn et al. 2022b). In addition, both religiosity and condom use were negatively related to mental health. These findings are consistent with the first and third tenets of CRT—*racism is endemic* and *differential racism*, respectively. As evidenced by endemic racism, we must ponder how discrimination, prejudice, and racism in the medical field results in both health and mental health inequalities that contributes to increased medical mistrust among people of color, including Black youth (Jaiswal and Halkitis 2019; Krull et al. 2020; Prather et al. 2018). Moreover, in terms of differential racism, health care practices may promote medical mistrust, which further contribute to disparate access to mental health and sexual health and reproductive information and resources based on race, as well as social class, i.e., racism (Gorry 2019; Krull et al. 2020). This implies that engaging Black youth in prosocial behaviors such as participating in responsible sexual activity—though powerful ways to influence their health equity must also be complemented by advocacy efforts to eliminate racism at the structural and systemic levels.

The multivariate analysis includes a statistically significant model that explains the associations between mental health and the study variables. We found strong positive and negative associations between Black youth and their mental health. Being an adolescent Black girl was associated with positive mental health, which is a noteworthy finding since they comprised 54% of the sample. In terms of CRT, the sixth tenet, *intersectionality/antiessentialism* focuses on different and multiple oppressions versus a sole focus on race and ethnicity and/or gender, which can overshadow other forms of exclusion, suffering, or fortitude. CRT theorists contend that analysis without a multidimensional framework can replicate the patterns of exclusion it seeks to combat and leads to the essentializing of oppressions (Hutchinson 2000). This is particularly relevant for Black girls as this sample was drawn from youth living in impoverished urban communities, yet they reported positive mental health. Quinn et al. (2019) noted the role of neighborhood cohesion among Black caregivers (mostly Black women) and how it buffered cultural race-related stress and further promoted non-threatening views of the police in their communities. Thus, being in a community with closely connected Black people could sustain and/or foster better mental health. In addition, family social support could bolster positive mental health outcomes (Brown 2008; Cooper et al. 2013; Gaylord-Harden et al. 2007; Smith et al. 2019), and given that considerable research suggests that higher perceived family social support was associated with decreased depression and anxiety, whereas less family social support was associated with the onset of depression among African American adolescents (Caldwell et al. 2002; Gaylord-Harden et al. 2007; Johnson and Kliewer 1999; Smith et al. 2019; Stice et al. 2004). However, the evidence is mixed as some scholars have noted that parents and socio-economic status (SES) could have the inverse effect. For example, a sample of African American girls involved with the youth punishment system (YPS) reported higher rates of posttraumatic stress disorder if they also reported parental support (Quinn et al. 2020, 2022a), while Blacks versus whites’ health and mortality have been less affected by SES (Assari 2015). Further, this finding is important because other studies suggest variations in results as another study with Black girls in a southern detention facility whose fear of condom use was associated with depressive symptoms (Waller et al. 2022). Rose et al. (2021) also conducted a study about African American and Caribbean girls’ mental health and religion, i.e., organized religious involvement (ORI), and they found that ORI was not directly related to mental health outcomes for African American girls. Our study results suggest that only the mental well-being and ORI for Black girls remained significant with religious and emotional support though they varied among the ethnic subgroups in the Rose et al. (2021) study. Further, *differential racialization* could explain this as Black girls reported mental well-being linked to ORI, which could be directly related to their involvement in the religious groups that could buffer the impact of racism and other power structures that tend to sustain their hardships.

Also noteworthy is that sex while on drugs and alcohol was positively associated with poorer mental health, which suggests that other factors could be relevant to this finding, for example violence and trauma. Research has established that mental health distress is linked to youth’s involvement with risky peers, substance misuse, and unsafe sexual practices (Lowe 2021; Voisin 2019). In focus groups with Black parents and caregivers on the Southside of Chicago, they noted how neighborhood violence affects the way youth experience sexual development and form romantic relationships, especially girls (Lowe 2021; Voisin 2019). Previous studies also indicate that Black youths’ mental health is related to the structural violence, systemic racism, and trauma they may regularly experience (Bath and Njoroge 2021; Meza and Bath 2021; Sheftall and Miller 2021; Voisin 2019). Furthermore, some scholars note that historical or ancestral trauma continues to impact the health, mental health, and healthcare decision-making of populations of color (Dorsey 2020; Quinn 2018; Sacks et al. 2021). Given that, we contend that Hypothesis 1 was partially supported.

To our surprise, all the other study variables: religiosity, attending church services, being in the 10th and 12th grades, and condom use, were all negatively associated with Black youths’ mental health. For example, religiosity and church attendance were negatively related to mental health. This is also important given that recent study findings with Black youth note their increased disengagement in S/R activities over time (Diamant 2018; Pew Research Center 2018). It is possible that the negative relationship between religiosity and mental health might be influenced by an intervening variable such as racism, which was not assessed in this study. Furthermore, it could be that youth attending church might actually report poorer mental health given that the topic of mental health is seldom addressed in Black churches. In addition, it is often assumed in Black churches that religious individuals should not have struggles with mental health if they have strong faith. Further, some Black youth who have experienced discrimination are more likely to meet the diagnostic criteria for a psychiatric disorder (M. O. Hope et al. 2017). Older youth, i.e., 10th and 12th graders, may be more affected by structural issues such as racism and/or discrimination in school settings, which could greatly influence their mental health. Boyd et al. (forthcoming) noted that Black youth who experienced racism in schools were much more likely to report suicidal behavior. CRT tenets such as *endemic racism* and *intersectionality* suggests the insidiousness of racism, along with the multiple and marginalized identities that impact Black youth and their mental health. Furthermore, the different reviews of empirical studies about the role of substance misuse and religiosity did not account for structural factors such as racism or discrimination that supports the need to incorporate CRT as a framework to address this gap.

Some scholars suggest that factors such as SES have a stronger effect on the health patterns of whites than Blacks, e.g., chronic disease (Assari 2015), depressive symptoms (Assari 2016), suicidality (Assari 2015), drinking patterns (Hummer and Lariscy 2011), and mortality (Assari and Lankarani 2016; Hayward et al. 2015). The reason(s) for this are unclear but could be due to the additional costs of upward mobility for Blacks versus whites (Hudson et al. 2020; Fuller-Rowell and Doan 2010; Fuller-Rowell et al. 2015), or heightened levels of racism against Blacks (Hudson et al. 2012) as SES produces less health for Blacks than whites (Assari 2018). Assari (2015) contends that social structure, segregation, and structural racism serve as mechanisms that promote health disparities of Black people, including Black youth. Thus, scholars have used Minorities’ Diminished Return Theory (Assari 2017, 2018) to reflect how societal, structural, and systemic barriers such as racism and discrimination hinder Black families and Black youth’s ability to make important health gains based on their increased income—SES resources (Assari 2018). In terms of CRT, the fourth tenet—*interest convergence/materialistic determinism* suggests that even when Black people achieve material advantage, which is often reserved for the majority race, i.e., white people, the advantages may have a limited affect. This results because true “progressive change” in terms of race can only occur when both groups’ interests—those of the dominant and the oppressed—converge (Bell 1995; Quinn and Grumbach 2015). Consequently, SES may have a weaker effect on the health of Blacks compared with whites (Assari et al. 2016; Montez et al. 2011), as the Minorities’ Diminished Return hypothesis has suggested (Assari 2017, 2018). As a result, Hypothesis 2 was partially supported.

### Limitations

Considering the findings within the current study’s limitations is essential. Our study findings may not be generalizable to all Black youth, given that this sample was drawn from one urban Midwestern city in the United States. The Black youth in the Resilience Project data were collected from the general population versus a selected or indicated population of youth with reported and/or clinically diagnosed mental health disorders. The study design is cross sectional so significant findings do not provide any temporal or causal inferences. In addition, the city of Chicago has received national attention due to violence, so our results may not be generalizable to youth who live in rural communities or have not been exposed to similar rates of violence, being houseless (Gabriel et al. 2022), or multi-system-involved (child welfare or youth punishment; Quinn et al. forthcoming b), as well as foreign-born youth who live in the United States—who may have been exposed to different types of violence, i.e., war and/or torture. CRT may shed light here as tenet four, *interest convergence/material determinism* focuses on the way “racism brings material advantage to the majority race, and progressive change regarding race occurs only when the interests of the powerful happen to converge with those of the racially oppressed” (Abrams and Moio 2009; Bell 1995; Quinn and Grumbach 2015, p. 6). Consequently, since the city has received national attention, there has been greater scrutiny placed upon Black versus white neighborhoods and residents, including an increase in resources, i.e., police presence, which could further penalize Chicago youth instead of addressing the long-standing inequities they face (Lowe 2021; Voisin 2019). Another limitation is that we did not include structural level variables to assess racism in our analytical models. CRT has three tenets that focus on racism and its construction and application in relationship to power and white supremacy in the United States, so though it is prudent to consider the role of protective variables such as S/R and how they could affect Black youths’ mental health the structural factors could influence their mental health in ways we have yet to examine. A final limitation is the use of single item variables and self-reported measures in our study. It may be useful to investigate multiple outcomes (i.e., sexual health and well-being) instantaneously within the same study population and design (VanderWeele 2017a, 2017b). Specifically, self-report has been noted for participant susceptibility to their responses being provided based on social desirability and recall biases, with potential resultant effects on our results.

Despite its limitations, our study has several strengths, including the focus on Black youth and their mental health, who remain an understudied and underserved population (Quinn et al. 2020). This is especially important given the recent and dramatic increase in Black youth and young adults dying by suicide (Boyd et al. 2022a, 2022b; Jones et al. 2021; Lindsey et al. 2019; Quinn et al. 2022b, 2022c). Our study findings suggest a need to focus on Black youth who reported lower (poorer) mental health, even if they also endorsed S/R.

Future research should continue to include S/R as it has long been a protective factor for youth during their formative years (Taylor et al. 2014), especially Black youth. Furthermore, much of the research in this area has been conducted with adults. This is the case even though research has begun to note that religious involvement may have positive (and diminishing) impacts on the health and well-being of youth, college students, and African Americans across the lifespan (Dupre et al. 2006; Ellison 1993; Ellison and Levin 1998; Idler 2014; Watson et al. 1988). Given this, future research needs to continue to focus on Black youth to address the gap our investigation sought to fill.

Studies with S/R as a protective factor may also need to disentangle spirituality from religiosity. Although scholars have delineated distinct differences in the two practices, spirituality is defined as faith or trust in the divine, peace, spiritual connectedness, and development that includes spiritual or religious coping, specifically prayer and meditation (Dill 2017). Spirituality is akin to religiosity and is often used interchangeably in the literature. However, some scholars could argue the religiosity variable in this study included factors that are more aligned with religiosity than spirituality. It also includes a fundamental belief in God, which most of our study sample endorsed. Moreover, religious involvement is also measured by prayer, church attendance, and meditation frequency. Future work should consider using more precise measures of both religiosity and spirituality. Conducting qualitative interviews or focus group discussions could help determine how Black youth view and categorize their own behavioral practices and establish relevant (and valid) measures for future investigations in larger samples of Black youth to assess their mental health.

Black youth who reported high levels of religious activity such as prayer and meditation could reflect their faith in God or a higher power even if they may not attend church services regularly. The negative relationship between religiosity and other variables such as condom use and mental health suggest a need to focus on measures of spirituality such as faith, as well as how youth’s belief in God or a higher power helps them to cope with personal hardships. Youth have started to engage in faith-based digital and social media communities for support versus organized religious involvement that could be associated with religious trauma resulting in negative mental health outcomes, as well as their disengagement in face-to-face activities such as in-person church attendance (Hill and Geldenhuys 2021). Similarly, adolescent spiritual development as evidenced in their hope, faith, and resilience (Benson et al. 2003) should be explored to assess shifts in critical consciousness that promote civic action to dismantle structural/systemic racism (Freeny et al. 2021; Palmer et al. 2021). In a qualitative study of Black college age women, Leath et al. (2022) noted that women at Historically Black Colleges and Universities had unique academic experiences that informed their critical consciousness^1^ and activism given the level of institutional support they received from faculty and administration in terms of organizing and leadership, as well as community care and involvement (Jean-Marie 2006). Critical consciousness could be used to help buffer the effects of racism, discrimination, and prejudice as noted by CRT. Moreover, it is an important part of sociopolitical development, a process that allows individuals to obtain the knowledge, analytical skills, emotional intelligence, and ability to effectively act in social and political structures and systems needed to identify, decipher, and defy oppression (Leath et al. 2022; Watts et al. 2003). Consequently, although traditional measures of religiosity may vary, by and large, African American youth continue to report “strong belief in the absolute truth of the Christian religion and scriptures” (Christerson et al. 2010, p. 140).

However, emerging evidence about Black youth’s S/R as a protective factor indicates that some forms of spirituality may be maladaptive. This is the case even though spirituality is a means of infusing the divine into the decision-making process (Edwards and Wilkerson 2018) and it can be used preventatively to guide their development of consequential thinking or reactively to provide insight on how to move beyond adverse interactions. Hardy et al. (2019) conducted a systematic literature review and found that some aspects of S/R are correlated with adverse outcomes, such as negative religious coping, controlled religious involvement, and religious doubt. These are maladaptive mechanisms that have led to internalizing negative affect, stress, increased substance misuse, and elevated sexual activity. Scholars also note that these conflicting findings regarding the role of S/R among Black youth are contextually specific and call for further examination. Furthermore, understanding the mental health and S/R needs of Black youth residing in areas with concentrated disadvantage is a first step to develop culturally salient supports and interventions. A systematic literature review notes that spirituality’s influence depends on contextual factors as we noted in our study results. Understanding how concentrated poverty and spirituality affect youth’s mental health decisions is pertinent to intervention development and delivery. In addition, the results from this study will help tailor interventions to enhance Black youth’s mental health outcomes. Research should include CRT to explore the structural, systemic, and societal issues (e.g., racism and poverty) that impact Black youth’s mental health. To that end, the results of our study may have important implications for designing future studies with longitudinal data to inform how S/R, drug use, and grade in school influence Black youth mental health over time. It is also essential to address self-report in future studies that could be improved by using technology to assist participants in answering questions (DiClemente et al. 2009). Utilizing mixed methodologies in future studies is also helpful in identifying participants’ views of the structural and systemic factors associated with their mental health such as violence, poverty, system involvement, and racism (excessive contact with law enforcement), to develop effective interventions that will help them to successfully navigate school settings and other aspects of their lives (M. O. Hope et al. 2017). Future research should consider investigating the protective factors that bolster their mental health, while also extrapolating the mixed results we found in this study. Further, using theories such as Minorities’ Diminished Return theory (Assari 2017), as well as CRT, could provide the needed multidimensional framework(s) to assess the unequal health gain(s) from SES as a key mechanism behind racial health disparities of Black youth. Multidimensional research examining individual and structural factors such as racism and discrimination is also warranted to promote full access to Black youth’s mental health needs. Further, ecodevelopmental theory has been useful in framing research studies investigating substance misuse, HIV transmission, and suicidal behavior (Quinn et al. 2022c; Szapocznik and Coatsworth 1999), including the protective roles of parents and peers (Bronfenbrenner 1979; Córdova et al. 2016; Boyd et al. 2022a, 2021; Quinn et al. 2022a, 2022b) with Black youth.

## 6. Conclusions

Presently, there is a heightened level of race-awareness in society, yet the task of demonstrating the challenges Black youth face to gain upward social mobility remains and is associated with more social, psychological, and physiological costs for Blacks than whites (Hudson et al. 2020; Fuller-Rowell and Doan 2010; Fuller-Rowell et al. 2015). Historically, religion has played a major role in the diverse stories of the black Diaspora in the United States (M. O. Hope et al. 2017). Our study suggests the need to include more macro-level factors such as racism and poverty, as well as Minorities Diminished Returns Theory, especially for youth in low-income settings in research studies. Evidence from our investigation extends the literature by delineating aspects of their lives that warrant greater investigation to address their mental health over their lifetimes. A lifecourse approach could produce significant knowledge about how to support youth at various stages of their development. Interventions should also comprise culturally tailored expertise about mental health equity to increase Black youth’s access to efficacious health and mental health services.

## Supplementary Material

supplementary material

## Data Availability

Data may be available upon request and approval of the last author.

## Footnotes

1 The process of developing one’s awareness of how their lived experiences reflect the broader cultural, political, and economic systems within United States society, which can also be harmful to people of color, i.e., Black girls and women (E. C. Hope et al. 2018)
