# Supplementary material for: The Relationship between Religion, Substance Misuse, and Mental Health among Black Youth
Source: Religions (Basel). Author manuscript; Available in PMC 2023 Nov 24. (PMC10673626; doi:10.3390/rel14030325)
Supplement: supplementary material [file NIHMS1943251-supplement-supplementary_material.pdf]

Table S1. Descriptive Statistics of study variables (N=636)		
**Variable**	**Frequency**	**%**
**Gender**		
Male	290	45%
Female	346	54%
**Age**		
12–14	118	19%
15–17	428	67%
18–22	89	14%
**Grade Levels**		
Freshman	200	32%
Sophomore	178	29%
Junior	117	19%
Senior	126	20%
**Sexual orientation**		
Heterosexual	475	81%
Gay	25	4%
Bisexual	59	10%
Pansexual	7	1%
Transgender	2	0.34%
Other	14	2.40%
**Importance of Religious/Spiritual Beliefs**		
Not Important	83	13%
Slightly important	101	16%
Moderately important	107	17%
Important	169	27%
Very important	159	26%
**Believe in GOD**	326	51%
Yes	578	93%
No	46	7%
**How often you Pray**		

Never	89	14%
Once in a while	244	29%
Fairly often	175	28%
Very often	119	19%
**Religious Service Attendance**		
Never	116	18%
Once in awhile	256	40%
Fairly often	129	20%
Very often	126	20%

Table S2. Substance Use variables (N=636)		
**Sex While on Drugs or Alcohol**		
Yes	44	12%
No	316	88%
**Drug Use**		
Marijuana Use		
Yes	250	40%
No	372	60%
Alcohol Use		
Yes	303	49%
No	321	51%
Ecstasy use		
Yes	26	5%
No	598	95%
Cigarette use		
Yes	82	13%
No	543	87%
Lean or Krokodil		
Yes	107	17%
No	515	82%
Crack or cocaine		
Yes	15	2%
No	607	98%

Table S3. Bivariate correlation study variables (N= 312)

Mental Health	1								
Sex while on Drugs or Alcohol	0.11*	1							
Drug Use	0.18***	0.30***							
Belief in God	-0.01	-0.06	1						
Religious/Spiritual Beliefs	0.03	-0.05	0.35***	1					
Pray or meditate	0.06	.00	0.29***	0.48***	1				
Religious service attendance	0.07	-0.03	0.19***	0.49***	0.47***	1			
Talk about religious concerns	0.12***	-0.02	0.13***	0.40***	0.48***	0.46***	1		
Gender	0.13***	-0.15***	0.08**	0.12***	0.09**	0.17***	0.01	1	
Condom use	-0.13	0.03	-0.01	0.04	-0.01	-0.02	0.06	-0.06	1

p<.05, p<.01**, p<.001****

Table S4. Multivariate Analysis on Mental Health (N=312)				
Variables	B	SE	P	β
Sex while on Drugs or Alcohol	5.19	2.44	0.04	0.12
Drug Use	2.00	0.45	0.01	0.24
Belief in God	1.27	2.67	0.63	0.03
Religious/Spiritual Beliefs	0.15	0.60	0.80	0.02
Pray or meditate	-0.50	0.89	0.58	-0.04
Religious service attendance	-0.40	0.87	0.64	-0.03
Talk about religious concerns	2.36	0.86	0.01	0.18
Gender	3.71	1.41	0.01	0.15
Condom Use	-3.14	1.48	0.04	-0.12
p<.05, p<.01**, p<.001****				
